# Association between dietary niacin intake and atherosclerotic cardiovascular disease among American adults: national health and nutrition examination survey

**DOI:** 10.3389/fnut.2025.1566684

**Published:** 2025-03-31

**Authors:** Yan Li, Beilei Yang, Na Li, Jinjuan Wei, Yue Wu

**Affiliations:** ^1^Department of Cardiology, Xi'an International Medical Center Hospital, Xi’an, Shaanxi, China; ^2^Department of Cardiovascular Medicine, People’s Hospital of Xiangxi Tujia and Miao Autonomous Prefecture, The First Affiliated Hospital of Jishou University, Jishou, China

**Keywords:** atherosclerotic cardiovascular disease, niacin, national health and nutrition examination survey, cross-sectional study, negative association

## Abstract

**Background:**

The relationship between dietary niacin and atherosclerotic cardiovascular disease (ASCVD) is still not fully understood. Our objective was to assess the association between dietary niacin intake and the prevalence of ASCVD.

**Methods:**

In this cross-sectional study, we examined a cohort of 15,685 adult individuals in the United States, aged 20 years and older, who participated in the National Health and Nutrition Examination Survey (NHANES) carried out between 2007 and 2014. Dietary Niacin consumption was assessed using a 24-h dietary recall method. The assessment of the presence of ASCVD was conducted through the Patient Medical Conditions Questionnaire. To assess the reliability of the results, restricted cubic spline models and logistic regression analyses were employed, along with conducting subgroup analyses.

**Results:**

The analysis included 15,685 participants who were 20 years or older, drawn from the NHANES data for the cycles spanning 2007 to 2014. Of which 10.4% (1,638/15,685) were diagnosed with ASCVD. The probability of ASCVD diminishes by 9% with each 10 mg/day increment in dietary niacin intake (OR = 0.91, 95% CI: 0.87–0.96). This association held true when niacin consumption was assessed as a categorical variable. Compared to individuals with the lowest dietary niacin intake, defined as T1 (≤17.4 mg/day), the adjusted odds ratios for ASCVD in those with higher niacin intakes, T2 (17.5–27.2 mg/day) and T3 (≥27.3 mg/day), were 0.87 (95% CI: 0.76–0.99, *p* = 0.037) and 0.75 (95% CI: 0.64–0.87, *p* < 0.001), respectively. There was an inverse association between dietary niacin intake and ASCVD prevalence, supported by sensitivity analyses. Subgroup analysis revealed an interaction effect when stratified by age.

**Conclusion:**

This analysis of NHANES data has demonstrated that niacin is significantly negative associated with ASCVD in American adults aged ≥20 years.

## Highlights

A large sample system was used to evaluate the dose–response association between dietary niacin intake and ASCVD. High dietary niacin intake is significantly associated with a reduced prevalence of ASCVD among American adults.

## 1 Introduction

The annual prevalence of atherosclerotic cardiovascular disease (ASCVD) has been increasing (1), exceeding the incidence rates of malignant cancers and several other systemic ailments. The death rate caused by ASCVD is also increasing in recent years, which brings great economic and physical harm to the population, and has attracted more and more attention. Previous studies have shown that the occurrence of new (ASCVD) is related to arteriosclerosis caused by dyslipidemia, hypertension, diabetes and other factors, of which hyperlipidemia is the main risk factor (2, 3). Once ASCVD is diagnosed, drug therapy is the most common treatment. When severe arterial stenosis occurred percutaneous arterial intervention and surgery were also be considered (4). In recent years, cardiac tissue engineering therapy targeting heart repair has also brought benefits (5). Recent research indicates a link between nutrients and patients with dyslipidemia (6). This information gives us potential targets for pharmaceutical and dietary strategies aimed at lowering the risk of ASCVD.

Niacin serves as a nutritional precursor for nicotinamide adenine dinucleotide (NAD) and nicotinamide adenine dinucleotide phosphate (NADP), both of which are essential cofactors involved in mitochondrial energy metabolism (7). Supplementation of niacin restores cellular NAD+ pool and enhances mitochondrial energy activities, while also reducing oxidative stress and the inflammatory response (8). Earlier research indicated that niacin is capable of reducing triglyceride levels by 25% while increasing HDL cholesterol by over 30% (9). Consequently, it has been utilized for many years to decrease triglycerides and enhance high-density lipoprotein (HDL) cholesterol. The deficiency of niacin in the diet may cause the increase of triglycerides and the decrease of high-density lipoprotein. According to previous clinical studies, oral or injected niacin can increase High Density Lipoprotein Cholesterol (HDL-C), thereby reducing the prevalence of ASCVD (10). However, few studies have investigated the relationship between dietary niacin and ASCVD in the general population.

The evidence in the association of niacin intake with ASCVD is insufficient, and there are also studies that dispute the relationship between them (11). The specific mechanism is not clear. In this study, we aimed to evaluate the association between dietary niacin and ASCVD using the data from the National Health and Nutrition Examination Survey (NHANES) (12). Considering the dietary habits observed in this group, we proposed that individuals with ASCVD are likely to consume less niacin in their diets.

## 2 Materials and methods

### 2.1 Study design and participants

This cross-sectional study used NHANES data from 2007 to 2014 (13). NHANES is a nationally representative survey conducted by the Centers for Disease Control and Prevention (CDC) and the National Center for Health Statistics (NCHS) in which stratified, multistage probability cluster sampling has been used to assess the health or nutritional status of the non-institutionalized US population (12). NHANES gathers extensive demographic and health-related data through home visits, screenings, and laboratory tests conducted by a mobile examination center (MEC). The survey’s thorough design, methodologies, and datasets are made available to the public. Ethical approval for NHANES was secured from the National Center for Health Statistics (NCHS) Ethics Review Committee, with all participants providing written informed consent prior to their involvement. Further secondary analysis did not necessitate additional approval from an Institutional Review Board. The NHANES data can be accessed via the NHANES website (retrieved on June 1, 2024). Our research focuses on participants aged 20 years and older who completed an interview. Data analysis was conducted from July to September 2024. We excluded pregnant women or individuals with missing data on atherosclerotic cardiovascular disease, dietary niacin intake, or covariates. This study adhered to the Strengthening the Reporting of Observational Studies in Epidemiology (STROBE) reporting guidelines.

### 2.2 Study variables and outcome

Our outcome is whether diagnosed with ASCVD. ASCVD includes acute coronary syndrome (ACS), those with a history of myocardial infarction (MI), stable or unstable angina or coronary or other arterial revascularization, stroke, transient ischemic attack (TIA), or peripheral artery disease (PAD) including aortic aneurysm, all of atherosclerotic origin (14).

We assessed whether a participant had been diagnosed with ASCVD by analyzing their responses to a question from the Medical Conditions Questionnaire: “Has a doctor or other health professional ever informed you that you had angina, a heart attack, stroke, or CHD?” During the NHANES dietary survey, participants were asked about the variety and amounts of foods and drinks they had consumed in the previous 24 h. Dietary intake information was collected from 2007 to 2014 through the NHANES Computer-Assisted Dietary Interview System (CADI), which utilizes a multiple-pass recall technique to provide interviewers with guidance on gathering food-related data. The United States Department of Agriculture gathered dietary consumption statistics through the Automated Multiple Pass Method (AMPM) during the same period. This fully computerized recall system formulates standardized queries and potential answers related to specific foods. By employing CADI and AMPM, we were able to accurately determine the nutritional values for each participant based on their food and beverage intake (15, 16). The NHANES Dietary Interviewers Procedure Manual offers a comprehensive outline of the methodology used in the dietary survey. We categorized the participants into three groups using the tertile approach according to their dietary niacin intake levels.

Covariate data were gathered through questionnaires, physical examinations, and laboratory testing. An array of potential covariates was evaluated based on existing literature (17) and clinical relevance, which included factors such as age, gender, race, education level, insurance status, smoking habits, physical activity, body mass index (BMI), hypertension, hyperlipidemia, diabetes, sleep difficulties, and dietary intake like energy, protein, carbohydrates, fats, niacin, and any dietary supplements take (17–19). In the NHANES study, race and ethnicity information was derived from survey questions regarding racial and Hispanic identity, classified into categories: non-Hispanic White, non-Hispanic Black, Mexican-American, and other racial groups. Educational levels were sorted into groups of less than 9 years, 9 to 12 years, and more than 12 years of education. Individuals possessing any form of medical insurance or health care plan(whether obtained via employment, direct purchase, or government programs like Medicare and Medicaid for medical assistance) were identified as having insurance coverage. Based on previous literature definitions, smoking status was divided into three groups: never smokers (those who have consumed fewer than 100 cigarettes), former smokers (who have quit after smoking over 100 cigarettes), and current smokers (17, 18). Physical activity is categorized into three types: sedentary, moderate, and vigorous. Moderate activity is defined as engaging in at least 10 min of movement during a typical week, which leads to a slight rise in either breathing or heart rate. In contrast, vigorous activity also involves at least 10 min of movement weekly, but it results in substantial increases in breathing or heart rate. Moreover, hypertension is characterized by a systolic blood pressure reading of 140 mmHg or higher and/or a diastolic reading of 90 mmHg or above, or a prior diagnosis of hypertension (20). The identification of other earlier diseases (such as diabetes, stroke, hyperlipidemia, and coronary artery disease) relied on responses from the questionnaire regarding whether the physician had been notified of these conditions previously. Body Mass Index (BMI) was calculated using a standardized method that took into account both weight and height (21). A dietary recall interview was conducted before the MEC interview to gather participants’ nutritional data for the past 24 h, which encompassed total caloric intake and fat consumption. Information about dietary supplements was acquired through inquiries about any nutritional supplements and medications taken in the previous month. Additional covariates considered include age, gender, sleep difficulties, and type of insurance. Details on all these variables can be found on the NHANES website, accessible at https://wwwn.cdc.gov/Nchs/Nhanes/continuousnhanes.

### 2.3 Statistical analysis

This study serves as a secondary analysis of datasets available to the public. The normality of continuous variables was verified using the Shapiro–Wilk statistical test. Proportions (%) were used to represent categorical variables. Continuous variables that followed a normal distribution were reported as mean (standard deviation, SD), while those with a skewed distribution were expressed as median (interquartile range, IQR). To assess group differences, we employed one-way analyses of variance for normally distributed data, Kruskal-Wallis tests for skewed data, and chi-square tests for categorical variables. Logistic regression models were applied to ascertain the odds ratios (OR) and 95 percent confidence intervals (95% CIs) concerning the connection between dietary niacin intake and the prevalence of ASCVD. We constructed 3 models: Model 1 was adjusted for sociodemographic characteristics, including age, gender, race and marital status. Model 2 was adjusted for sociodemographic characteristics and the factors that *p* values were less than 0.01 in the univariate analysis (including PIR, educational attainment, smoking status, physical activity, dietary supplements taken, insurance and BMI),and Model 3 was fully adjusted, including Mode 2 and hyperlipemia, diabetes mellitus, hypertension and trouble sleeping.

Furthermore, a restricted cubic spline (RCS) regression analysis was conducted using four knots at the 5, 35, 65, and 95 percentiles of dietary niacin. This approach was utilized to evaluate linearity and analyze the dose–response relationship between dietary niacin and ASCVD, following the adjustments of variables outlined in Model 3.

Additionally, the potential changes in the association between dietary niacin and ASCVD were evaluated, taking into account the following factors: age (<65 years vs. ≥65 years), sex, race (non-Hispanic White compared to non-Hispanic Black and Mexican-American or other racial groups), marital status (cohabiting with partners vs. living alone), educational attainment (<9 years vs. 9–12 years or more than 12 years), body mass index (BMI) (<25 kg/m^2^ vs. 25–30 kg/m^2^ or over 30 kg/m^2^), sleep disorders, and physical activity levels (sedentary vs. moderate or vigorous). Variability across subgroups was analyzed using multivariate logistic regression, and interactions within subgroups were examined through the likelihood ratio test.

Since the determination of the sample size relied solely on the existing data, no preliminary calculations for statistical power estimates were carried out. All analyses utilized the statistical software packages R version 4.2.2 (The R Foundation, Shanghai, China; accessed on 10 July 2024)^2^ and Free Statistics software version 2.0 (Beijing, China).^3^ A descriptive analysis was performed involving all participants. A two-tailed test was employed, with a *p*-value of less than 0.05 considered indicative of statistical significance.

## 3 Results

### 3.1 Study population

**Figure 1:**
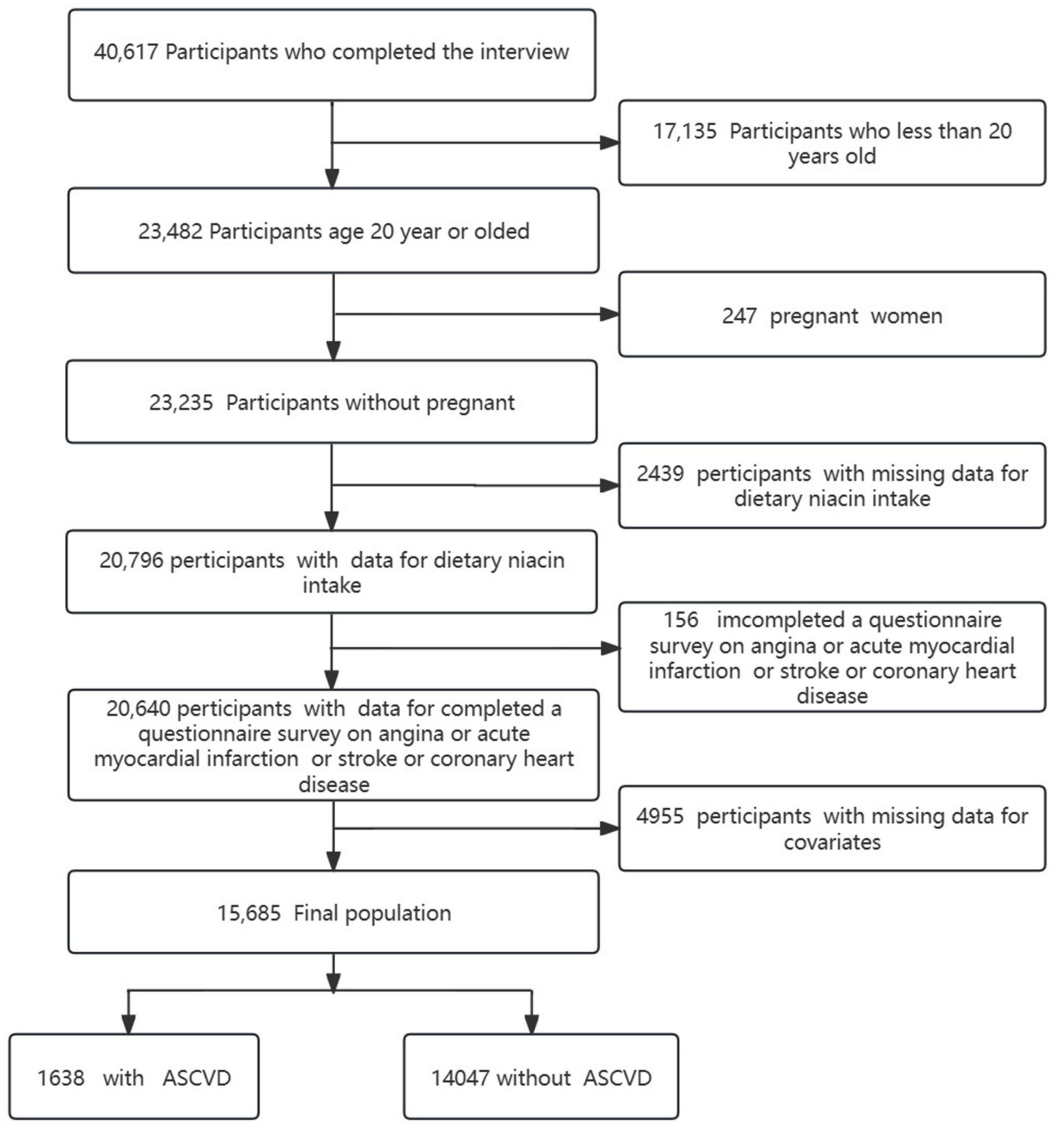
Flowchart of the sample selection from NHANES 2007–2014.

A total of 40,617 participants completed interviews, of whom 17,135 were excluded for younger than 20 years old and 156 were missing data for ASCVD (including coronary heart disease, angina, heart attack, and stroke). The missing data of pregnant women (*n* = 247), the missing data of dietary niacin intake (*n* = 2,439) and the missing data of covariates (*n* = 4,955) were all excluded. Ultimately, 15,685 participants were involved in this cross-sectional study from NHANES between 2007 and 2014. The prevalence of ASCVD in the participants was 10.4% (1,638/15,685). The detailed inclusion and exclusion process is shown in Figure 1.

### 3.2 Baseline characteristics

The baseline characteristics of all study subjects according to their dietary niacin intake tertiles was described in Table 1. There were 1,638(10.4%) patients with ASCVD. The mean age of the participants was 51.1 (17.2) years old, of which 8,111 (51.7%) individuals were female. There were significant differences in niacin intake among all groups for age and sex (*p* < 0.01). Individuals with a higher intake of niacin tended to be female, Mexican American, had a higher educational level, non-smoking, with any insurance, sleep accessibility, had a lower prevalence of hypertension, hyperlipidemia, and diabetes. However, niacin intake was higher in sedentary people compared with moderate or vigorous activity.

**Table 1:** Baseline characteristics of the study participants.

Characteristic	Niacin intake,(mg/d)				*p*-value
	Total	T1 (≤17.4)	T2 (17.5–27.2)	T3 (≥27.3)	
No.	15,685	5,227	5,229	5,229	
Age(year), Mean ± SD	51.1 ± 17.2	54.2 ± 17.5	51.8 ± 17.0	47.4 ± 16.5	< 0.001
Sex, n (%)					< 0.001
Male	7,574 (48.3)	1,656 (31.7)	2,364 (45.2)	3,554 (68)	
Female	8,111 (51.7)	3,571 (68.3)	2,865 (54.8)	1,675 (32)	
Married Status, n (%)					< 0.001
Married or living with a partner	9,467 (60.4)	2,938 (56.2)	3,251 (62.2)	3,278 (62.7)	
Living alone	6,218 (39.6)	2,289 (43.8)	1978 (37.8)	1951 (37.3)	
Race/ethnicity, n (%)					< 0.001
Non-Hispanic White	7,441 (47.4)	2,325 (44.5)	2,530 (48.4)	2,586 (49.5)	
Non-Hispanic Black	3,364 (21.4)	1,217 (23.3)	1,084 (20.7)	1,063 (20.3)	
Mexican American	1902 (12.1)	648 (12.4)	605 (11.6)	649 (12.4)	
Others	2,978 (19.0)	1,037 (19.8)	1,010 (19.3)	931 (17.8)	
Education level (year), n (%)					< 0.001
<9	1,351 (8.6)	638 (12.2)	389 (7.4)	324 (6.2)	
9–12	5,596 (35.7)	1959 (37.5)	1852 (35.4)	1785 (34.1)	
>12	8,738 (55.7)	2,630 (50.3)	2,988 (57.1)	3,120 (59.7)	
PIR, n (%)					< 0.001
Low	4,805 (30.6)	1859 (35.6)	1,518 (29)	1,428 (27.3)	
Medium	5,664 (36.1)	1946 (37.2)	1883 (36)	1835 (35.1)	
High	5,216 (33.3)	1,422 (27.2)	1828 (35)	1966 (37.6)	
Smoking status, n (%)					< 0.001
Never	8,635 (55.1)	2,976 (56.9)	2,900 (55.5)	2,759 (52.8)	
Former	4,055 (25.9)	1,255 (24)	1,411 (27)	1,389 (26.6)	
Current	2,995 (19.1)	996 (19.1)	918 (17.6)	1,081 (20.7)	
Physical activity, n (%)					< 0.001
Sedentary	8,020 (51.1)	2,950 (56.4)	2,729 (52.2)	2,341 (44.8)	
Moderate	4,418 (28.2)	1,492 (28.5)	1,526 (29.2)	1,400 (26.8)	
Vigorous	3,247 (20.7)	785 (15)	974 (18.6)	1,488 (28.5)	
Coronary heart disease, n (%)	696 (4.4)	262 (5)	252 (4.8)	182 (3.5)	< 0.001
Angina, n (%)	436 (2.8)	170 (3.3)	160 (3.1)	106 (2)	< 0.001
Heart attack, n (%)	690 (4.4)	276 (5.3)	242 (4.6)	172 (3.3)	< 0.001
Stroke, n (%)	620 (4.0)	294 (5.6)	187 (3.6)	139 (2.7)	< 0.001
Cholesterol, n (%)	6,164 (39.3)	2,159 (41.3)	2,103 (40.2)	1902 (36.4)	< 0.001
Diabetes, n (%)	2,132 (13.6)	843 (16.1)	721 (13.8)	568 (10.9)	< 0.001
BMI(kg/m^2^), Mean ± SD	29.4 ± 7.0	29.6 ± 7.1	29.4 ± 6.9	29.1 ± 7.0	< 0.001
Calorie consumption(kcal/d), Median (IQR)	1906.0 (1416.0, 2553.0)	1363.0 (1040.0, 1723.0)	1916.0 (1556.0, 2357.0)	2647.0 (2100.0, 3355.0)	< 0.001
Protein consumption(g/d), Median (IQR)	73.2 (52.0, 99.5)	47.4 (35.5, 59.3)	73.7 (60.5, 88.4)	109.0 (88.8, 137.9)	< 0.001
Carbohydrate consumption(g/d), Mean ± SD	252.4 ± 124.6	184.1 ± 83.3	244.5 ± 95.0	328.6 ± 141.6	< 0.001
Fat consumption(g/d), Median (IQR)	70.0 (46.9, 100.7)	49.3 (33.6, 68.1)	71.2 (51.3, 95.4)	99.3 (70.6, 134.2)	< 0.001
Fiber consumption(g/d), Median (IQR)	14.5 (9.6, 21.4)	10.5 (6.9, 15.5)	14.6 (10.3, 20.4)	19.4 (13.6, 27.1)	< 0.001
Dietary Supplements taken, n (%)	6,583 (42.0)	2,245 (43)	2,246 (43)	2092 (40)	0.002
Sleep disorder, n (%)	1,503 (9.6)	519 (9.9)	498 (9.5)	486 (9.3)	0.536
Insurance, n (%)	12,742 (81.2)	4,261 (81.5)	4,312 (82.5)	4,169 (79.7)	0.001
ASCVD, n (%)	1,638 (10.4)	679 (13)	549 (10.5)	410 (7.8)	< 0.001

PIR, Ratio of family income to poverty; BMI, body mass index; CHD, Coronary Heart Disease; DM, diabetes; AMI, heart attack; ASCVD, Atherosclerotic Cardiovascular Disease.

### 3.3 Association between dietary niacin intake and ASCVD

The univariate analysis indicated that every covariate had an association with ASCVD. Specifically, factors such as age, race, body mass index (BMI), hypertension, hyperlipidemia, diabetes mellitus (DM), sleep disturbances, history of smoking or current smoking status, and the consumption of dietary supplements were all positively associated with ASCVD (Supplementary Table S1).

**Figure 2:**
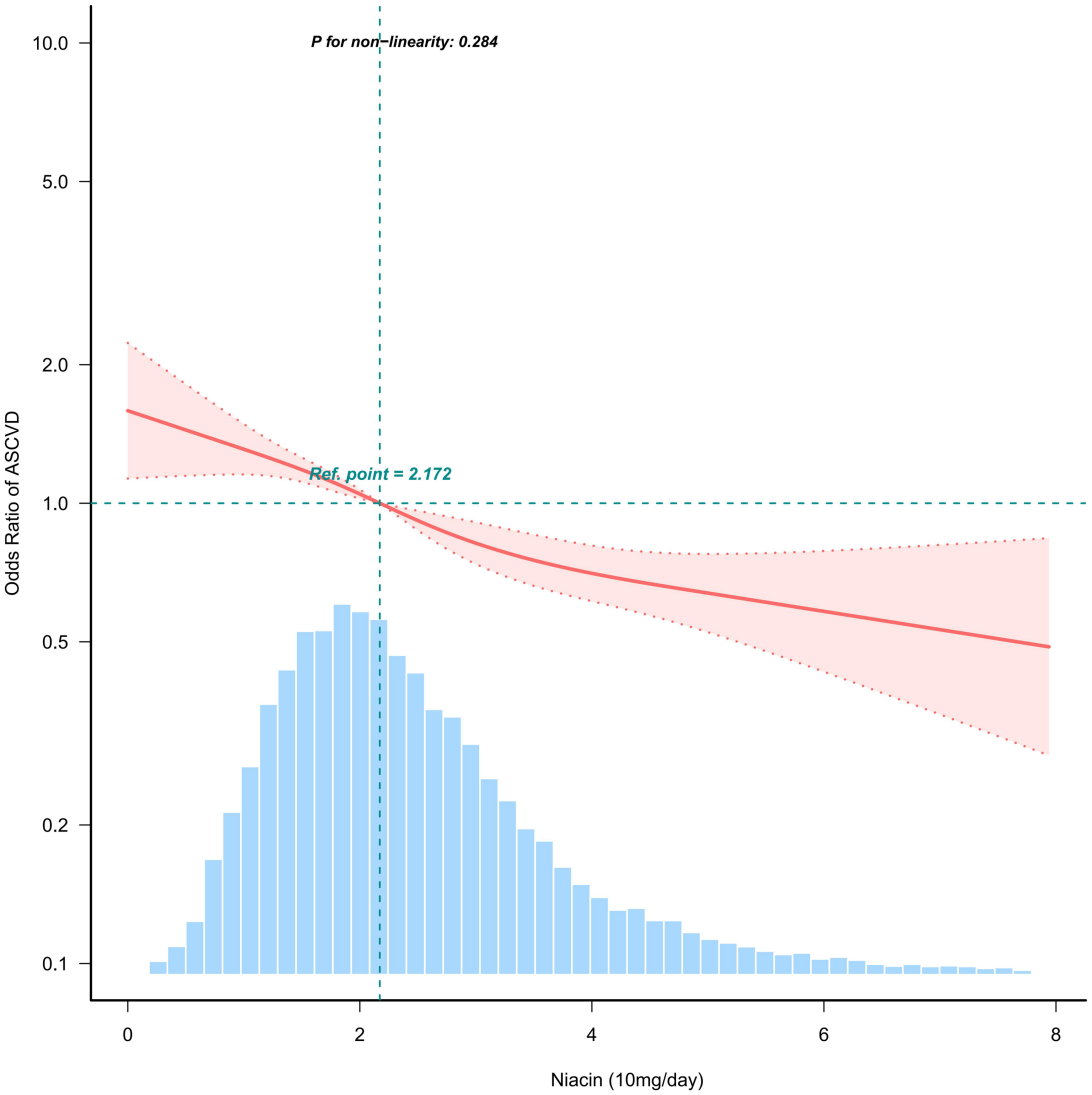
Association between dietary niacin intake and ASCVD. Solid and dashed lines represent the predicted value and 95% confidence intervals. They were adjusted for age, sex, marital status, ratio of family income to poverty, education status, smoking status, physical activity, dietary supplements taken, insurance, body mass index, cholesterol, hypertension, diabetes and sleep disorder. Only 99% of the data is shown.

When dietary niacin intake was analyzed using tertiles, after adjustment for potential confounders, dietary niacin intake was generally inverse association with the prevalence of ASCVD. Compared to individuals with lower niacin intake T1 (≤17.4 mg / day), the adjusted OR values of dietary niacin intake and ASCVD in T2 (17.5–27.2 mg / day) and T3 (≥27.3 mg / day) were 0.87 (95% CI: 0.76–0.99, *p* < 0.05) and 0.75 (95% CI: 0.64–0.87, *p* < 0.001), respectively (Table 2). Accordingly, dietary niacin intake was negative associate with the prevalence of ASCVD (*p* = 0.284) in RCS (Figure 2).

**Table 2:** Association between dietary niacin intake and ASCVD.

Variable	N. total	N. event_%	Crude		Model 1		Model 2		Model 3	
			OR (95%CI)	*p*	OR (95%CI)	*p*	OR (95%CI)	*p*	OR (95%CI)	*p*
Dietary niacin (10 mg/day)	15,685	1,638 (10.4)	0.83 (0.8–0.87)	<0.001	0.9 (0.85–0.94)	<0.001	0.92 (0.87–0.96)	<0.001	0.91 (0.87–0.96)	<0.001
Tertile										
T1 (≤17.4)	5,227	679 (13)	1(Ref)		1(Ref)		1(Ref)		1(Ref)	
T2 (17.5–27.2)	5,229	549 (10.5)	0.79 (0.7–0.89)	<0.001	0.83 (0.73–0.94)	0.005	0.88 (0.77–1)	0.057	0.87 (0.76–0.99)	0.037
T3 (≥27.3)	5,229	410 (7.8)	0.57 (0.5–0.65)	<0.001	0.7 (0.6–0.8)	<0.001	0.76 (0.66–0.88)	<0.001	0.75 (0.64–0.87)	<0.001
P for Trend				<0.001		<0.001		<0.001		<0.001

T, tertile; OR, odds ratio; CI, confidence interval; Ref, reference; DM, diabetes; PIR, ratio of family income to poverty; BMI, body mass index. Model 1: Age + Sex + Race + Marital status. Model 2: Model 1 + PIR + Education status + Smoke + Physical activity + Dietary supplement + Insurance + BMI. Model 3: Model 2 + Cholesterol + Hypertension + DM + sleep disorder.

### 3.4 Stratified analyses based on additional variables

**Figure 3:**
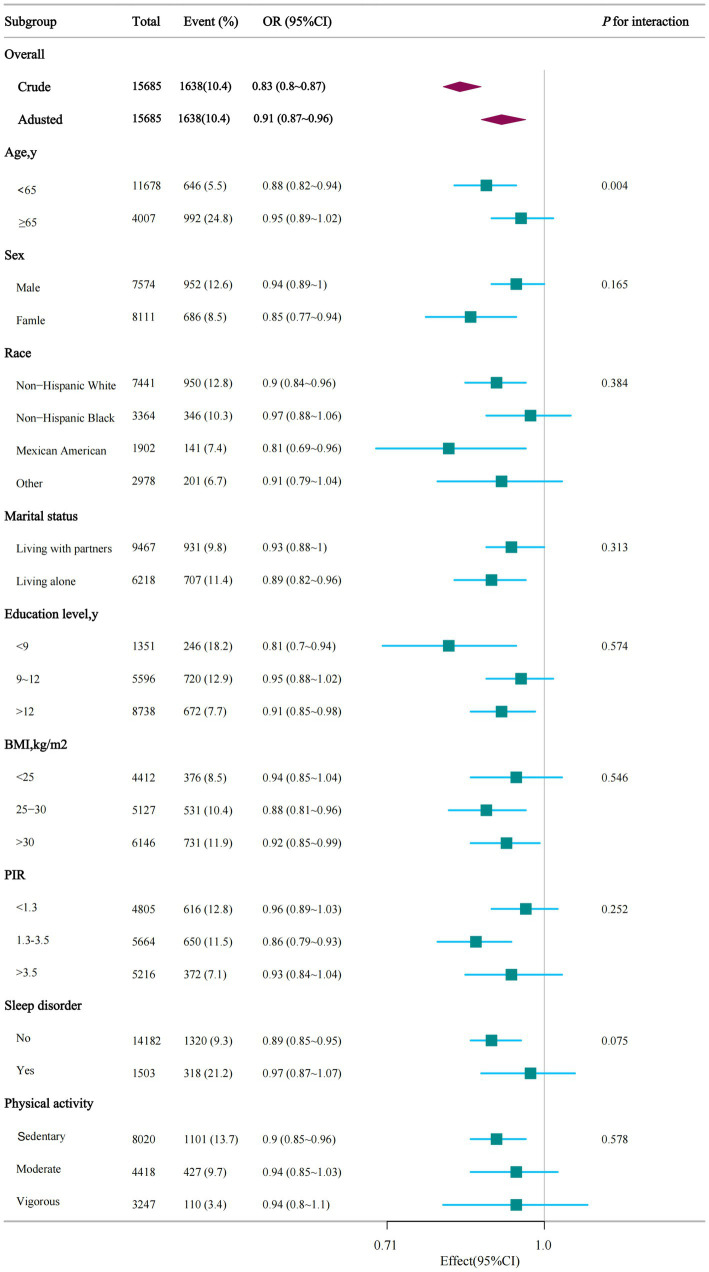
Stratified analysis of dietary niacin intake and ASCVD. PIR, ratio of family income to poverty; BMI, body mass index.

In multiple subgroups, a stratified analysis was conducted to evaluate possible effect modifications concerning the connection between dietary niacin and ASCVD. The analysis produced consistent findings across different strata, and no significant interactions were identified in any subgroups when categorized by sex, race, marital status, education level, family income, BMI, sleep disorders, and physical activity (Figure 3). Taking into account the issue of multiple testing, a *p*-value below 0.05 for the age interaction may not hold statistical significance.

### 3.5 Sensitivity analysis

After removing individuals with excessively high energy intake, a total of 15,383 participants remained, and the relationship between dietary niacin intake and ASCVD continued to be consistent. In comparison to those with lower niacin intake in T1 (≤17.4 mg/day), the adjusted odds ratio (OR) values for dietary niacin intake and ASCVD in T2 (17.5–27.2 mg/day) and T3 (≥27.3 mg/day) were found to be 0.86 (95% CI: 0.75–0.99, *p* = 0.033) and 0.69 (95% CI: 0.59–0.81, *p* < 0.001), respectively (Supplementary Table S2).

## 4 Discussion

This large cross-sectional study of American adults showed a negative association between dietary niacin intake and ASCVD, with the prevalence of ASCVD decreasing as dietary niacin intake increased.

Recently, various dietary modification methods have been used in many studies to try to reduce the prevalence of ASCVD. It seems that the prevalence of ASCVD can be reduced by modifying the diet. Some researchers had found that there is an association between serum iron and ASCVD, and the serum iron levels were negatively associated with ASCVD in the US elderly population (21). Also, there are other researchers had found that the dietary vitamin K levels were negatively associated with ASCVD in the US elderly population (18). However, few researches have assessed the association between dietary niacin intake and the onset of ASCVD, especially in the United States population. Niacin serves as a crucial nutritional precursor for both nicotinamide adenine dinucleotide (NAD) and nicotinamide adenine dinucleotide phosphate (NADP), essential cofactors involved in the energy metabolism of mitochondria (22). Niacin is nutrient fortified in food staples (23). Foods rich in niacin include liver, chicken, turkey, ground beef, fish and brown rice (24), its supplementation is considered a method to increase NAD+ levels in the body, but its role in ASCVD is not well understood. In a study by Emily Wuerch, researchers proposed that niacin is an essential nutrient for the treatment of pellagra (25). In the follow-up studies, niacin was reported to improve vascular endothelial function by suppressing oxidative stress (26), and niacin can help manage cholesterol levels in the blood. Therefore, before the advent of statins, niacin was commonly used to treat dyslipidemia (27). Many studies have explored the effect of niacin on patients with coronary heart disease (CHD), and have demonstrated the efficacy of niacin in reducing the chance of cardiovascular events and mortality (28). Niacin’s influence on CVD has ever been attention in some other studies, in a recent study provided the evidence that higher dietary niacin intake resulted in reduced risk of all-cause and cardiovascular disease mortality among CVD patients (29). Cynthia Tannous’s team highlight the significance of preserving NAD equilibrium in various models of heart diseases (30). Adding niacin to statin therapy has not been shown to further decrease ASCVD events (31), but this result is controversial. In Stanley L. Hazen’s study, through an untargeted metabolomics analysis of fasting plasma from stable cardiac patients in a prospective discovery cohort, it suggested that niacin metabolism was associated with incident major adverse cardiovascular events (MACE) (23). Xuyang Geng’s study showed that plasma niacin is inversely associated with hyperlipidemia in participants with diabetes among Chinese adults (32). A recent study by Shi Su showed that Nanoparticle-Directed antioxidant therapy can ameliorate disease progression in a novel, diet-inducible model of coronary artery disease (33). It is likely that niacin intake may be beneficial to the cardiovascular health. The objective of our research was to explore the connection between dietary niacin and ASCVD within the general population of the United States. The NHANES provides a distinctive opportunity to evaluate the potential association between dietary niacin intake and ASCVD, as well as to assess the dose–response association, while thoroughly accounting for various covariates and conducting a variety of stratified analyses. Our study shows that dietary niacin intake is independently associated with a reduced prevalence of ASCVD.

Dietary niacin intake may reduce the prevalence of ASCVD through several mechanisms. However, the preventive role of dietary niacin supplements in the prevalence of ASCVD has not well be clarified. In Marc Ferrell’s study, they found that a terminal metabolite of niacin promotes vascular inflammation and contributes to cardiovascular disease risk (23). It is well established that prolonged or extensive exposure to oxidized phospholipids (OxPLs) can lead to persistent inflammation (34). OxPLs are found in atherosclerotic lesions and can be detected in plasma on lipoproteins that contain apolipoprotein B (apoB). A strong correlation exists between plasma OxPL-apoB measurements and levels of lipoprotein(a) (Lp(a)) in plasma. Both experimental and clinical studies have demonstrated that among apoB-containing lipoproteins, Lp(a) particles carry the highest proportion of OxPLs. The levels of plasma OxPL-apoB offer crucial diagnostic insights regarding the presence and severity of atherosclerosis, enhancing the ability to prognosticate peripheral artery disease as well as the risk of first and recurrent myocardial infarctions and strokes. Furthermore, plasma OxPL-apoB levels are predictive of cardiovascular events with accuracy comparable to or exceeding that of plasma Lp(a) levels, as this measurement accounts for both genetic factors contributing to elevated Lp(a) levels and the oxidative modifications affecting apoB-containing lipoproteins that incite inflammation. Treatment methods such as Lp(a)-lowering therapy with antisense oligonucleotides, lipoprotein apheresis, niacin therapy, and bariatric surgery have been shown to decrease plasma OxPL-apoB levels (35).

Several limitations must be taken into account. To begin with, ASCVD data were collected exclusively from NHANES during the period from 2007 to 2014. This restriction hindered our ability to utilize NHANES data from various time frames for additional validation. Additionally, although regression models and stratified analyses were conducted, it remains impossible to completely rule out residual confounding effects resulting from unmeasured or unknown factors. Finally, the present findings originate from a survey involving adults in the United States, and further research is needed to determine if these results can be applied to other populations. Fourth, dietary intake of niacin was assessed through a 24-h recall method, which could introduce recall bias. In contrast, the food frequency questionnaire offers less specific information regarding types and amounts of food compared to the 24-h recall. Ultimately, because of the fundamental limitations of cross-sectional studies, the causal link between niacin and ASCVD remains undetermined and should be validated through longitudinal research in the future. Beyond the association between nutrition and ASCVD, we may also investigate additional lifestyle factors that could influence ASCVD in future studies, including physical activity and others.

## 5 Conclusion

In conclusion, higher dietary niacin intake group was associated with a lower chance of developing ASCVD. After sensitivity analysis, the results were still robust. This study’s findings highlight the association between ASCVD and the intake of dietary niacin.

## Data Availability

The datasets presented in this study can be found in online repositories. The names of the repository/repositories and accession number(s) can be found at: https://wwwn.cdc.gov/nchs/nhanes/default.aspx 2007–2014.

## Footnotes

^2^ http://www.R-project.org

^3^ http://www.clinicalscientists.cn/freestatistics
