# Supplementary material for: Association between dietary niacin intake and atherosclerotic cardiovascular disease among American adults: national health and nutrition examination survey
Source: Front Nutr. 2025 Mar 31;12:1566684. doi: 10.3389/fnut.2025.1566684 (PMC11994425; doi:10.3389/fnut.2025.1566684)
Supplement: Supplementary file 1 [file Table_1.DOCX]

Supplementary Table S1 Association of covariates and ASCVD risk.

| Variable | OR(95%CI) | *P*-Value |
| --- | --- | --- |
| Age(year) | 1.07 (1.07~1.08) | <0.001 |
| Sex,Female | 0.64 (0.58~0.71) | <0.001 |
| Education level(year) |  |  |
| <9 | 1(Ref) |  |
| 9-12 | 0.66 (0.57~0.78) | <0.001 |
| >12 | 0.37 (0.32~0.44) | <0.001 |
| Race/ethnicity |  |  |
| Non-Hispanic White | 1(Ref) |  |
| Non-Hispanic Black | 0.78 (0.69~0.89) | <0.001 |
| Mexican American | 0.55 (0.45~0.66) | <0.001 |
| Others | 0.49 (0.42~0.58) | <0.001 |
| PIR |  |  |
| Low | 1(Ref) |  |
| Medium | 0.88 (0.78~0.99) | 0.036 |
| High | 0.52 (0.46~0.6) | <0.001 |
| BMI(kg/m^2^) | 1.02 (1.01~1.03) | <0.001 |
| Fiber consumption | 0.98 (0.98~0.99) | <0.001 |
| Niacin consumption | 0.98 (0.98~0.99) | <0.001 |
| Zinc consumption | 0.99 (0.98~0.99) | 0.001 |
| Vitamin k consumption | 1 (1~1) | <0.001 |
| Married Status |  |  |
| Married or living with a partner | 1(Ref) |  |
| Living alone | 1.18 (1.06~1.3) | 0.002 |
| Smoking status |  |  |
| Never | 1(Ref) |  |
| Former | 2.56 (2.28~2.88) | <0.001 |
| Current | 1.79 (1.56~2.06) | <0.001 |
| Physical activity |  |  |
| Sedentary | 1(Ref) |  |
| Moderate | 0.67 (0.6~0.76) | <0.001 |
| Vigorous | 0.22 (0.18~0.27) | <0.001 |
| Hypertension | 5.81 (5.16~6.54) | <0.001 |
| Diabetes | 4.01 (3.57~4.5) | <0.001 |
| Sleep disorder | 2.61 (2.28~3) | <0.001 |
| Insurance | 2.5 (2.1~2.98) | <0.001 |
| Dietary Supplements taken | 1.63 (1.47~1.81) | <0.001 |

**PIR**: Ratio of family income to poverty; **BMI:** body mass index.

Supplementary Table S2 Association between dietary niacin intake and ASCVD (excluding the extreme energy intake)

| **Variable** | **N.total** | **N.event_%** | **Crude** | | **Model1** | | **Model2** | | **Model3** | |
| --- | --- | --- | --- | --- | --- | --- | --- | --- | --- | --- |
|  |  |  | OR (95%CI) | *P* | OR (95%CI) | *P* | OR (95%CI) | *P* | OR (95%CI) | *P* |
| Dietary niacin  (10mg/day) | 15383 | 1638 (10.4) | 0.82 (0.78~0.86) | <0.001 | 0.84 (0.8~0.88) | <0.001 | 0.87 (0.83~0.91) | <0.001 | 0.87 (0.83~0.92) | <0.001 |
| Quartile |  |  |  |  |  |  |  |  |  |  |
| Q1(≤17.4) | 5128 | 660 (12.9) | 1(Ref) |  | 1(Ref) |  | 1(Ref) |  | 1(Ref) |  |
| Q2(17.5-27.2) | 5127 | 539 (10.5) | 0.8 (0.7~0.9) | <0.001 | 0.83 (0.73~0.94) | 0.005 | 0.87 (0.76~0.99) | 0.037 | 0.86 (0.75~0.99) | 0.033 |
| Q3( ≥ 27.3) | 5128 | 406 (7.9) | 0.58 (0.51~0.66) | <0.001 | 0.62 (0.54~0.72) | <0.001 | 0.69 (0.59~0.8) | <0.001 | 0.69 (0.59~0.81) | <0.001 |
| P for Trend |  |  |  | <0.001 |  | <0.001 |  | <0.001 |  | <0.001 |

**Q,** quartile; **OR,** odds ratio; **CI,** confidence interval; **Ref,** reference; **DM,** diabetes; **PIR,** ratio of family income to poverty; **BMI, b**ody mass index

**Model 1:** Age+Sex+Race+Marital status

**Model 2:** Model 1+PIR+Education status+Smoke+Physical activity+Dietary supplement+Insurance+BMI

**Model 3:** Model 2**+C**holesterol+Hypertension+DM+Sleep disorder
